# Effects of Exogenous 6-Phytase (EC 3.1.3.26) Supplementation on Performance, Calcium and Phosphorous Digestibility, and Bone Mineralisation and Density in Weaned Piglets

**DOI:** 10.3390/ani11061787

**Published:** 2021-06-15

**Authors:** Núria Tous, Joan Tarradas, Maria Francesch, Maria Font-i-Furnols, Peter Ader, David Torrallardona

**Affiliations:** 1 Animal Nutrition, Institut de Recerca i Tecnologia Agroalimentàries (IRTA), Centre Mas Bové, Ctra. Reus-El Morell km. 3.8, 43120 Constantí, Spain; nuria.tous@irta.cat (N.T.); joan.tarradas@irta.cat (J.T.); maria.francesch@irta.cat (M.F.); 2 Product Quality and Technology, Institut de Recerca i Tecnologia Agroalimentàries (IRTA), Finca Camps i Armet, 17121 Monells, Spain; maria.font@irta.cat; 3 BASF SE, Animal Nutrition, Europe. Chemiestrasse 22, 68623 Lampertheim, Germany; peter.ader@basf.com

**Keywords:** bone density, computed tomography, phosphorous digestion, phytase, pigs

## Abstract

**Simple Summary:**

Supplementation with a low dose (up to 500 FTU/kg feed) of a new 6-phytase, obtained by assembling gene sequences of various phytase-producing bacteria, increases phosphorous and calcium availability in phosphorus-limiting piglet diets. Therefore, this phytase can be used to reduce the use of unsustainable sources of inorganic phosphorous without compromising piglets’ performance and bone mineralization and density, and the higher phosphorous utilization will also reduce its excretion to the environment. This study also demonstrates that the effects of phytase on bone density can be evaluated using computed tomography, which may be useful for studies with live animals.

**Abstract:**

Phosphorus (P) is an essential mineral for growing piglets, which is poorly accessible in vegetable feedstuffs as it is stored as phytates. Thus, phytase supplementation is essential to increase P availability. Two experiments were conducted to evaluate a novel 6-phytase (EC 3.1.3.26) in weaned pigs fed low-P diets. In each experiment, one hundred and twenty piglets were fed a positive control (PC; adequate in Ca and P), a negative control (NC; limiting in Ca and P), or NC supplemented with 125, 250, or 500 FTU/kg of phytase (NC125, NC250, and NC500, respectively). P content was lower in diets of Experiment 1 than diets of Experiment 2. In Experiment 1, piglets offered PC or phytase diets had higher growth and efficiency compared with NC diets. In Experiment 2, similar effects were obtained, but the effects were less significant. In both experiments, P and Ca ATTD and bone density were significantly increased with phytase supplementation. Moreover, PC and NC500 had higher P concentrations and lower alkaline phosphatase activity in plasma than NC. To conclude, supplementation with the new 6-phytase at doses up to 500 FTU/kg enhanced P utilization, growth performance, and bone density in piglets fed P-limiting diets.

## 1. Introduction

Phosphorus (P) is an essential mineral, necessary for bone mineralization and for the regulation of key enzymes in several metabolic and physiological processes in growing animals [1]. In plant seeds and grains, this element is mostly stored in the salt form of phytic acid (phytate), myo-inositol hexaphosphate (IP6) being the predominant form, and this is the main source of P for animal nutrition [2,3]. The production of endogenous phytase by pigs is limited, and these animals are not able to hydrolyse enough phytate-bound P to satisfy their nutritional requirements [4,5,6].

Phytic acid also has several antinutritional effects that have been reviewed recently [7]. Due to its large chelating capacity, phytic acid can interact with minerals (calcium [Ca], sodium, iron, and zinc) and other substances (amino acids, starch, and lipids) and compromise their absorption [8,9,10,11,12]. Furthermore, as phytate can also bind to proteins, it could inhibit the activity of digestive enzymes (i.e., trypsin, α-amylase, and pepsin) and impair digestibility [13,14]. As a result, phytates in animal feed are responsible for reduced nutrient absorption, limited energy availability, and reduced animal growth [15]. Moreover, pigs may excrete up to 50–80% of P intake [16], and this is considered to have a major environmental impact on soils and water [17,18].

In order to reduce these effects, the supplementation of feeds with exogenous microbial phytases has become a common practice [19]. Phytases have a specific affinity for IP6 and myo-inositol pentaphosphate (IP5). The formation of lower phosphorylated and more soluble inositol phosphates (IP5, IP4) by the action of phytase improves the susceptibility to further enzymatic hydrolysis and accelerates further degradation to IP3 and IP2, as lower phosphorylated inositol phosphates show less mineral binding capacity [20]. Therefore, phytases increase the available P, reducing the phytate excretion and increasing digestibility [21,22].

Exogenous phytases are usually of microbial origin, and they can be classified as 3-phytases (EC 3.1.3.8) or 6-phytases (EC 3.1.3.26), depending on the position at which the hydrolysis of the phytate molecule is initiated (C3 or C6, respectively). Besides type of activity, the efficacy of phytases depends on many factors, including microorganism of origin, optimal pH and temperature, type of substrate or species or age of the animals [15,23], and specific pattern of potential P-hydrolysis from phytic acid via various inositol phosphate isomers to myo-inositol (IP0) [24]. Recently, a newly developed composited bacterial 6-phytase has been tested, obtaining promising results [25]. The novelty of that phytase is that the phytase gene was assembled from sequences of various phytase-producing bacteria (i.e., *Hafnia ssp*., *Yersinia ssp.*, and *Buttauxiella ssp.*). The production was done by fermentation in *Aspergillus niger*.

The evaluation of phytase efficacy varies depending on the measurement used for its assessment. Performance and P apparent total tract digestibility (ATTD) are probably the most commonly used, but bone breaking strength, bone mineralization, and probably bone density are the most sensitive criteria of P availability [26]. Dual-energy X-ray absorptiometry (DXA or DEXA) is the technique of choice to determine bone density [27]. It allows the evaluation of thick body parts or complex bone formations, because it obviates the need for assumptions about soft-tissue shape and attenuation [28]. On the other hand, quantitative computed tomography (CT) is the only three-dimensional densitometer available, capable of selectively measuring cortical or trabecular bone [29]. Computed tomography has also been used to determine bone density, studying the surface with certain Hounsfields values [30,31,32]. Both DXA and CT have the advantage that allows the study of bone characteristics non-destructively, either applied to live animals or carcasses or directly to the bones.

Two experiments were conducted with the objective of evaluating the efficacy of different doses of this new phytase on performance, bone mineralization, and ATTD of Ca and P in diets formulated with low digestible P fed to weaned piglets. Measurement of bone density using CT was also evaluated as a possible tool to evaluate the efficacy of phytases in piglet feeding.

## 2. Materials and Methods

The two experiments were conducted at the Experimental Farm of the “Institut de Recerca i Tecnologia Agroalimentària–IRTA” at Centre Mas Bové, Constantí (Spain).

### 2.1. Animals, Dietary Treatments, and Experimental Design

One hundred and twenty newly weaned pigs ([Duroc × Landrace] × Pietrain; mixed entire males and females) were used in each of the two experiments. Piglets had average ages of 26 and 28 days and body weights of 7.3 ± 1.37 and 9.2 ± 1.24 kg (mean ± SD) at the start of Experiments 1 and 2, respectively. Pigs in each experiment were allotted to a randomized complete block design with body weight as the blocking factor. There were five experimental treatments consisting of a positive control diet (PC) that was formulated to contain adequate levels of Ca and P, a basal negative control diet (NC) that was formulated to contain limiting levels of Ca and P, and the basal NC diet supplemented with either 125, 250, or 500 FTU/kg of the novel phytase under study (NC125, NC250, and NC500, respectively). The phytase activity (FTU/kg) of diets NC125, NC250, and NC500, analysed according to Engelen et al. [33], were 96, 266, and 422 (pre-starter; Experiment 1); 100, 253, and 577 (pre-starter; Experiment 2); 111, 223, and 454 (starter; Experiment 1); and 106, 245, and 469 (starter; Experiment 2), respectively. The PC diets were formulated to provide 0.36% and 0.29% digestible P (ATTD) in the pre-starter and starter period, respectively, according to NRC [34] for Experiment 1 and 0.38% and 0.33% digestible P according to FEDNA [35] for Experiment 2. This meant that higher digestible P contents were used in Experiment 2 compared to Experiment 1. For the basal NC diets, digestible P contents were reduced by half by reducing the amount of dicalcium phosphate in the diets. The total Ca:P ratio was maintained at 1.2 for all diets. Besides Ca and P, PC and NC diets were formulated to be iso-nutritive for each phase in both experiments. Each experimental treatment comprised eight replicates with three piglets per pen. Pigs had ad libitum access to feed and water according to a 2-phase feeding program: pre-starter phase (14.1 MJ ME; 12.5 g/kg SID lysine) between 0–21 days, and starter phase (14.0 MJ ME; 11.5 g/kg SID lysine) between 21–42 days of the experiment. The composition of the diets and their analysed nutrient composition are shown in Table 1. Feed was presented in pelleted form, and phytase was added in a liquid formulation by post-pelleting liquid application. Titanium dioxide (5 g/kg) was added to the pre-starter phase experimental diets as a marker for digestibility measurements.

### 2.2. Experimental Procedures, Sampling, and Analyses

Individual BW and feed intake for each pen were recorded at days 0, 21, and 42, and the pen’s average initial and final live body weight (BW), average daily weight gain (ADG), average daily feed intake (ADFI), and average gain to feed ratio (GFR) between 0–21, 21–42, and 0–42 days of the experiment were calculated. Between days 18–21, fresh faeces from each pen were collected and stored at −20 °C until freeze-drying and analysis. Feed and faeces were analysed for Ca, P, and TiO_2_. Calcium was analysed by inductively coupled plasma-optical emission spectrometry (ICP-OES Optima 2100DV, Perkin Elmer Life and Analytical Sciences, USA). Total P was determined colorimetrically by the vanadomolybdate method-AOAC 965.17 (AOAC, 2000), and the concentrations of TiO_2_ were measured as described by Short et al. [36]. The ATTD coefficients for Ca and P were calculated by the index method [37].

At the end of each experiment (d 42), the piglets with the intermediate initial body weight in each pen (one piglet per pen) were slaughtered under isofluorane anesthesia. After confirmation of death, the left front foot of the piglets was sampled and stored at −20 °C until analysis. Feet were defrosted overnight and autoclaved at 105 °C for 15 min, after which the *Os metacarpale III* and *IV* were dissected. *Os metacarpale III* was used for the analysis of dry matter and ash content, and *Os metacarpale IV* was reserved for CT scanning. For the piglets belonging to treatments PC, NC, and NC500, a blood sample was obtained before slaughtering using lithium heparin tubes. Calcium (o-cresolphthalein complexone method) and phosphate (ammonium-molybdate reaction method) concentrations and AP activity (IFCC method, 37 °C) in blood plasma were analysed with a Roche Hitachi 917 clinical chemistry analyser (Roche Diagnostics, Basel, Switzerland).

### 2.3. Computed Tomography Scanning of the Bones

**Figure 1:**
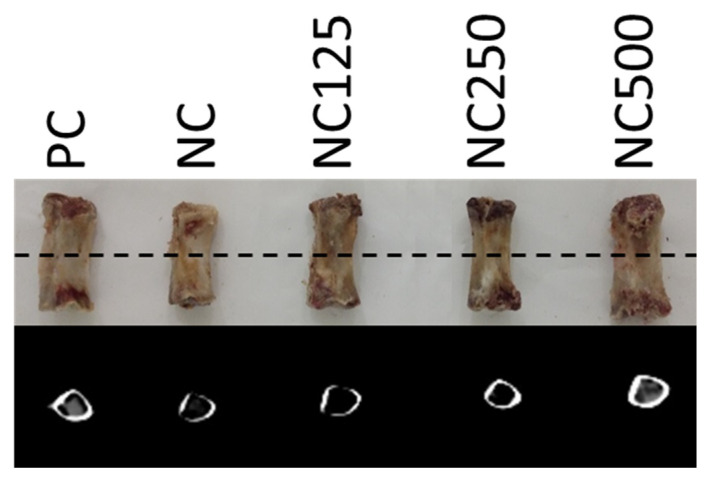
Example of disposition of the bones (one of each treatment) for the computed tomography (CT) acquisition and axial image obtained of the centre of the bones (discontinuous line).

The *Os metacarpale IV* bones were scanned by CT in groups of five bones (one from each treatment) according to the initial blocks of BW. The bones from the five piglets in each block were placed in parallel on the CT table, and an axial image was taken from the centre of the bone by means of a General Electric HiSpeed Zx/l CT device (GE Healthcare, Boston, Massachusetts, USA). The following acquisition parameters were used: 140 kV, 145 mA, 512 × 512 matrix, 1 mm-thick image, and a displayed field of view of 350 mm. Figure 1 shows an example of the disposition of the bones and the axial image obtained with CT.

From the axial images of the bones, the volumes associated with each Hounsfield unit (HU) were obtained individually for each bone by means of the software VisualPork, developed by the University of Girona and IRTA [38,39]. Bones were considered to have HU values higher than 140 [40], and the volume associated with these HU values was obtained. This limit also allows the discarding of the marrow of the bone analysis, since it has lower density [41,42], and the consideration of only the cortical bone. This volume for each axial image and the distribution of volumes for each bone were used to determine bone density according to the formula developed by Picouet et al. [43]. In addition, bone tissues were classified between those that have low density (HU values between 141 and 500) and those that have high density (HU values above 500), as proposed by du Chazaud et al. [30] and applied in other works [31]. The proportion of low (volume between HU 141 and 500 with respect to total bone, i.e., HU > 140) and high-density (volume with >500 with respect to total bone, i.e., HU > 140) bone tissues was calculated as a measure of bone strength.

### 2.4. Statistics

The effects of dietary treatment were analysed by ANOVA using the GLM procedure of SAS (SAS Inst. Inc., Cary, NC, USA) whereby the pen was considered as an experimental unit. The following model was used:

*Y_ij_* = *μ* + *τ_i_* + *β_j_* + *e_ij_* (1)

where *Yij* is the observed dependent variable, *μ* is the overall mean, *τ_i_* is the effect of dietary treatment, *β_j_* is the effect of block, and *e_ij_* is the random error within the model. All the animals completed the experiments satisfactorily, and their data has been included in the calculations and the statistical analysis, and no outliers were detected according to Smirnoff-Stefansky’s test [44]. Linear and quadratic polynomial contrasts were also used to determine the response to phytase supplementation of the NC diets. When the effect of the dietary treatment was significant, multiple comparisons of treatment means were performed using Fisher’s LSD test. The level of significance was set at *p* < 0.05, and trends are discussed at *p* < 0.1.

## 3. Results

### 3.1. Performance

Growth performance parameters are shown in Table 2. In Experiment 1, no statistically significant differences in performance among treatments were observed during the pre-starter phase (0–21 days), except for GFR, which tended to be lower in piglets fed the NC diet than in those fed the NC125 diet (*p* = 0.06). A quadratic response to the addition of phytase to the NC diets was also observed for GFR in the pre-starter phase (*p* < 0.05). During the starter phase (21–42 days) piglets fed the NC diet had significantly lower ADG and ADFI than any of the other treatments (PC or phytase-supplemented NC diets) (*p* < 0.01). No differences were observed between PC and the NC diets supplemented with phytase. Linear responses to increasing doses of phytase were observed for ADG and ADFI (*p* < 0.01). Finally, during this phase, the GFR of the animals fed the NC diet was lower than that of the animals fed the PC and NC500 diets (*p* < 0.05), and for the NC supplemented diets, a linear increase with the dose of phytase was observed (*p* < 0.05). Over the whole experiment (0–42 days), piglets fed the NC diet had significantly lower final BW and ADG than those fed the PC or any of the phytase-supplemented diets (*p* < 0.01), lower GFR than those fed the PC, NC125, and NC500 diets (*p* < 0.01), and smaller ADFI than those fed the PC and NC500 diets (*p* < 0.05). Linear responses to the dose of phytase were observed for final BW, ADG, and ADFI (*p* < 0.01).

In Experiment 2, the performance responses to the different dietary treatments were in line with those of Experiment 1, although no significant differences were reached for most of them. Only ADG of pigs fed the NC diet during the starter phase was significantly lower than that of pigs fed the PC, NC250, or NC500 diets (*p* < 0.05). Pigs fed the NC diet also tended to have lower final BW and poorer ADG over the whole experiment than the pigs fed PC or NC250 diets (*p* < 0.10) and lower GFR during the starter phase than the pigs fed PC or NC500 diets (*p* < 0.10). Linear responses to the dose of phytase added to the NC diets were observed for ADG (*p* < 0.05) and GFR (*p* < 0.01) during the starter phase.

### 3.2. Apparent Total Tract Digestibility of P and Ca

The ATTD of P and Ca are shown in Table 3. In Experiment 1, ATTD of P for the NC diet was significantly lower than that of the PC and all the phytase-supplemented diets (*p* < 0.01). Linear (*p* < 0.01) and quadratic (*p* < 0.05) responses were also observed to the increasing doses of phytase on the NC diet. Furthermore, Ca ATTD of the NC diet was not different from that of the PC diet, but it was enhanced by phytase supplementation relative to NC (all doses) or PC (250 and 500 FTU doses; *p* < 0.01). Linear and quadratic responses to the dose of phytase on the NC diet were observed for Ca ATTD (*p* < 0.01).

In Experiment 2, P ATTD of the NC diet was significantly lower than that of the PC and the NC250 and NC500 supplemented diets (*p* < 0.01), and a linear (*p* < 0.01) response to the dose of phytase on the NC diet was observed. Ca ATTD of the NC diet was also lower than that of the PC, NC250, and NC500 diets (*p* < 0.01), and it augmented linearly (*p* < 0.01) and quadratically (*p* < 0.01) with increasing doses of phytase on the NC diet.

### 3.3. Bone Mineralization and Bone Density

The parameters of bone mineralization and density are shown in Table 4. In Experiment 1, *Os metacarpale III*’s dry weight and ash content (both expressed as percentage and as total weight) and bone density of *Os metacarpale IV* were lower for the NC diets than for the PC diets (*p* < 0.01), and relative to NC, phytase supplementation increased bone dry weight and total ash content for all phytase containing diets (*p* < 0.01), and ash percentage and bone density for NC250 and NC500 diets (*p* < 0.01).

In Experiment 2, *Os metacarpale III*’s dry weight and ash content (both, expressed as percentage and as total weight) were lower for the NC diets than for the PC diets, and relative to NC, phytase supplementation increased bones’ dry weight for diets NC250 and NC500 (*p* < 0.01); ash percentage for NC500 (*p* < 0.01), and total ash content for all phytase containing diets (*p* < 001). No significant differences in bone density of *Os metacarpale IV* were observed for this experiment.

Significant linear responses to the doses of phytase in the NC diet were observed in both experiments for bone’s dry weight and total ash content (*p* < 0.01) and for ash percentage (*p* < 0.01). In Experiment 1, linear and quadratic responses were also observed for bone density (*p* < 0.01).

### 3.4. Image Analysis of Bones by Computed Tomography Scanning

**Figure 2:**
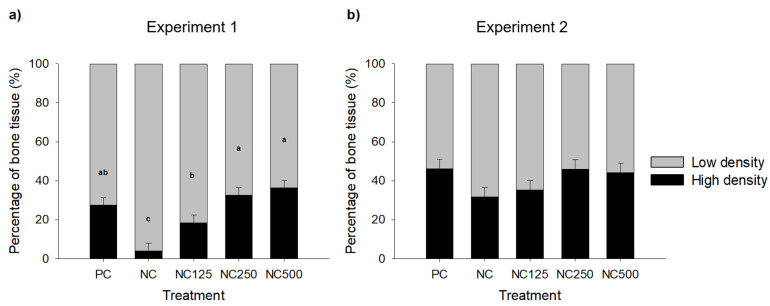
Proportion of low-density (HU values ranging from 141–500) and high-density (HU values above 500) tissues with respect to total bone (HU > 140) in left *Os metacarpale IV* bone of piglets as measured by computed tomography in (**a**) Experiment 1 and (**b**) Experiment 2. NC = negative control; NC125 = NC diet with phytase at 125 FTU/kg feed; NC250 = NC diet with phytase at 250 FTU/kg feed; NC500 = NC diet with phytase at 500 FTU/kg feed. Values in the table are LSMEANS of one animal per pen (8 pens per treatment) for each experiment. Bars with different letters differ significantly at *p* < 0.05 (LSD test). Significant linear and quadratic responses to phytase dose addition to the NC diet were observed in Experiment 1 (*p* < 0.001 and *p* = 0.016, respectively).

Figure 2 shows the proportion of the *Os metacarpale IV* bones with tissue of low density (HU values between 141 and 500) and tissue of high density (HU values above 500) in Experiments 1 (Figure 2a) and 2 (Figure 2b). In Experiment 1, pigs fed the NC diets presented a lower proportion of high-density bone tissue compared to PC (*p* < 0.01). Phytase supplementation also increased the proportion of bone tissue with high density compared to NC, and this was significant for all the phytase diets (*p* < 0.05). The proportion of high-density bone tissue also increased linearly (*p* < 0.01) and quadratically (*p* < 0.05) with the dose of phytase. Similar patterns were observed for Experiment 2, but no significant effect was observed. However, a tendency for a linear response to phytase supplementation was detected (*p* = 0.06).

### 3.5. Concentration of Ca and P and Alkaline Phosphatase Activity in Blood Plasma

The concentrations of Ca and P and the activity of AP in blood plasma are reported in Table 5. No statistically significant effects of dietary treatment on Ca plasma levels were observed in any of the two experiments. In both experiments, relative to PC and NC500 diets, the NC diets resulted in reduced concentrations of P (*p* < 0.01), increased Ca:P ratios (*p* < 0.01), and increased AP activity (*p* < 0.01 and *p* < 0.05 for Experiments 1 and 2, respectively).

## 4. Discussion

It is well known that a large amount of P from plant-based diets is not available for monogastric animals, because it is found as a phytate form [2]. The addition of phytases to the diets favours P hydrolysis and may also increase the digestibility and absorption of other nutrients and performance [15,45,46,47].

This study showed that phytase supplementation of NC diets resulted in values of body weight, weight gain, feed intake, and gain/feed ratio that were equivalent to those observed with the corresponding PC diets. These performance parameters were significantly improved in the phytase-supplemented diets compared to NC, and linear dose effects were observed. These significant improvements in performance with the addition of phytase were observed mainly during the starter phase (21–42 d), but no statistically significant differences were reached during the pre-starter phase (0–21 d after weaning). Other authors from similar studies have also reported less statistical significance for performance parameters for the immediate period after weaning. Bento et al. [48] conducted two experiments of 24 days (animals from 8 to 12 kg) and observed that *Buttiauxella* phytase supplementation (at 250, 500, 1000, or 2000 FTU/kg) did not have an effect on the performance parameters, despite increasing Ca and P digestibility. In another experiment with weaned piglets, 1000 FTU/kg of phytase did not have a significant effect on performance during the first 21 days of the experiment, but significant improvements were found at day 35 [49]. A study with an *E. coli*-derived phytase in weaning pig diets over 28 days indicated that phytase supplementation improved growth performance and Ca and P digestibility in pigs during the starter phase [50]. In a recently published meta-analysis [51], it has been estimated that phytase supplementation increases P digestibility by an average of 25.6% digestibility units. This metanalysis identified diet composition, animal’s age, and phytase characteristics as the main factors influencing the efficiency of phytases. In the current studies, the differences in phytase efficiency observed between the pre-starter and starter phases could be explained by a higher variability in the immediate period post-weaning, associated with post-weaning stress [52], but also by a limited ability of the piglets during this period to secrete HCl and acidify the stomach contents to pH values that optimise the activity of the phytase. Whereas the pH in the stomach of young piglets remains close to that of the diet, in the adult pig, pH rapidly falls to values close to pH 2 [53]. In weaned piglets, it has been described that the pH of digesta in the fundus region of the stomach of piglets was close to 3.5 at weaning and during the first week postweaning, and it was not until the second week post-weaning that average pH values below 3 were achieved [54].

On the other hand, it is well recognised that exogenous phytases increase nutrient digestibility and reduce the antinutritional effects of phytates, both effects contributing to enhancing animal performance and reducing the impact of animal production on the environment [15,25]. In the case of P, the key issue is how much additional P is potentially released and made available to the animals, which mainly depends on the composition of the diet. However, the impact on performance and bone mineralisation also depends on how well the basal control diet meets the P requirements of the piglets. In the current experiments, at day 21 post-weaning, phytase supplementation of low-P diets with the 500 FTU increased P digestibility by up to 32% and 18% in digestibility units, respectively. In a previous experiment with the same phytase and dosage, an increase of 37% in digestibility units and an additional release of 1.57 g of P per kg were observed [25]. An average response to phytase supplementation of 19% units of P digestibility has been reported for piglets [55], which indicates a high efficacy for the novel phytase tested. It should be noted that P digestibility in the current study is very low in the NC diet (particularly in Experiment 1). According to the exponential prediction model proposed by Sung et al. [56], ATTD P contents of 1.079 and 1.663 g/kg should be expected for the NC pre-starter diets in Experiments 1 and 2, respectively. Our results were much lower than expected with this prediction model, as for these diets, we obtained values of 0.304 and 1.164 g ATTD P/kg, respectively. This could be explained by the low P digestibility of corn diets in young pigs, as has been previously reported [57,58].

Serum AP activity is negatively correlated with bone mineralisation and has been used as an indicator for Ca and P status in pigs [5,59,60]. In the present experiments, the pigs consuming the NC diet presented lower P concentration and higher PA activity in blood serum than pigs from the PC group or pigs fed the NC diet supplemented with 500 FTU of phytase, which confirms a shortage of available P with the NC diet but adequate and equivalent levels for the PC and NC supplemented diets. This agrees with other studies in which a higher availability of P resulting from phytase supplementation caused a reduction in serum PA activity [61,62,63,64]. It is worth noticing that lower values of serum PA activities and higher P concentrations were found in Experiment 2 than in Experiment 1. These may be explained by a higher P availability in the basal diets of Experiment 2, resulting from the addition of an additional 0.24% of dicalcium phosphate.

Several authors have used bone mineralisation as an adequate indicator of P status [26,65,66]. Many studies have observed a positive response in bone mineralization to phytase supplementation of a low-P diet, providing similar values to those obtained with P-adequate diets [54,67,68]. With the phytase evaluated in the present work, the bone mineralization response, in terms of changes in overall bone density, was maximised with supplementation doses ranging between 250 and 500 FTU/kg of phytase, which are lower activities than those reported in previous studies to achieve the same effect [26,62,68,69,70,71,72]. In Experiment 1, a linear increase in bone density to phytase dose was also observed, but this effect was not as clear in the piglets of Experiment 2. This is probably because of the different P contents of the basal diets. In Experiment 1, the highest bone density was 1.62 g/cm^3^, and this was observed for the highest dose of phytase, the PC being 1.56 g/cm^3^, whereas in Experiment 2, the highest bone density was 1.72 g/cm^3^ for the 250 FTU dose of phytase, a value that was very similar to that observed for the 125 and 500 FTU doses and the PC (ranging from 1.68 to 1.71 g/cm^3^), which suggests that the maximal response may have already been achieved with the lower doses of phytase. In a similar study with fattening pigs, it has been shown that supplemental inorganic P can be completely removed from the diet without affecting performance if phytase is added to the diet [26]. These authors tested the morphometric, geometrical, densitometric, and mechanical properties of the pig’s femur at the end of the study. The results showed that the strength and bone density (measured by dual-energy X-ray absorptiometry) of the pigs fed an inorganic-P free diet and supplemented with 1175 FTU of phytase were better than those of the pigs fed the control diet supplemented with 5.04 g monocalcium phosphate per kg. It demonstrates that, if enough substrate (phytate) is available, phytase may satisfy P requirements. In the present studies, it was also observed that supplementing low-P diets with phytase resulted in equivalent bone densities to those obtained with diets adequate in P.

## 5. Conclusions

The supplementation of low-P diets with the novel bacterial 6-phytase tested at dose rates up to 500 FTU/kg improves performance, phosphorous digestibility, and bone mineralization and density in piglets fed P-limiting diets, and these effects were more evident for the basal diet with lower P content. In addition, it has also been shown that bone density evaluated by computed tomography can be used to test the phytase efficacy in piglets.

## Figures and Tables

**Table 1:** Composition and nutrient content of basal diets ^1^.

	1–21 d (Pre-Starter)				21–42 d (Starter)			
	Experiment 1		Experiment 2		Experiment 1		Experiment 2	
	PC	NC	PC	NC	PC	NC	PC	NC
Ingredients (%)								
Maize	48.4	50.5	48.1	50.1	65.9	67.5	64.9	66.6
Soybean meal (48% CP)	24.2	24.2	24.4	24.4	27.8	27.8	28.5	28.5
Extruded soybeans	10.0	10.0	10.0	10.0	-	-	-	-
Sweet milk whey	11.0	11.0	11.0	11.0	-	-	-	-
Lard	2.69	1.86	2.75	1.91	2.83	2.14	2.89	2.20
L-Lysine-HCl	0.32	0.31	0.31	0.31	0.38	0.37	0.38	0.37
L-Threonine	0.13	0.13	0.13	0.12	0.15	0.14	0.15	0.15
DL-Methionine	0.18	0.17	0.18	0.17	0.15	0.15	0.15	0.14
L-Tryptophan	0.03	0.03	0.03	0.03	0.03	0.03	0.03	0.03
Salt	0.16	0.16	0.16	0.16	0.36	0.36	0.36	0.36
Calcium carbonate	0.38	0.55	0.21	0.38	0.45	0.59	0.28	0.42
Dicalcium phosphate ^2^	1.39	0.05	1.63	0.29	1.41	0.30	1.65	0.54
Ethoxyquin ^3^	0.02	0.02	0.02	0.02	0.02	0.02	0.02	0.02
Vit-Min complex ^4^	0.60	0.60	0.60	0.60	0.60	0.60	0.60	0.60
TiO_2_	0.50	0.50	0.50	0.50	-	-	-	-
Analysed nutrient content (%)								
Dry matter	89.7	89.3	88.1	88.0	89.0	88.4	87.4	87.3
Crude Protein	21.0	20.1	20.5	20.9	19.0	19.0	19.4	19.1
Fat	6.06	5.31	7.77	6.40	5.58	4.91	5.31	4.32
Ash	6.14	5.02	5.68	4.86	4.92	4.11	4.66	3.97
Gross Energy (MJ/kg)	17.4	17.3	17.0	16.9	17.3	17.1	16.9	16.7
Calcium	0.83	0.55	0.81	0.47	0.78	0.60	0.67	0.57
Phosphorous	0.57	0.31	0.66	0.40	0.56	0.38	0.63	0.44

PC = positive control; NC = negative control. ^1^ Calculated phytate-P levels for both experiments were 0.23% (PC) and 0.24% (NC) in pre-starter diets and 0.24% (PC) and 0.25% (NC) in starter diets. ^2^ DCP had a phosphorous content of 17.5%, and digestible P content from that source was assumed to be 13.5%. ^3^ Provides per kg feed: vitamin A 10,000 UI; vitamin D_3_ 2000 UI; vitamin E (alfa-tocopherol) 25 mg; vitamin B_1_ 1.5 mg; vitamin B_2_ 3.5 mg; vitamin B_6_ 2.4 mg; vitamin B_12_ 20 µg; vitamin K_3_ 1.5 mg; calcium pantothenate 14 mg; nicotinic acid 20 mg; folic acid 0.5 mg; biotin 50 µg; Fe (FeSO_4_·H_2_O) 120 mg; I (Ca(I_2_O_3_)_2_) 0.75 mg; Cu (CuSO_4_·5H_2_O) 150 mg; Mn (MnO) 60 mg; Zn (ZnO) 110 mg; Se (Na_2_SeO_3_) 0.37 mg. ^4^ Antioxidant 6-ethoxy-1,2-dihydro-2,2,4-trimethylquinoline (Andrés Pintaluba S.A., Spain).

**Table 2:** Effect of 6-phytase supplementation to negative control diets on performance of weaned pigs ^1^.

	PC	NC	NC125	NC250	NC500	*RMSE*	*p-*Value	*L* ^2^	*Q* ^3^
Experiment 1									
* Body weight (kg)*									
0 d	7.33	7.37	7.30	7.33	7.29	0.090	0.414	0.145	0.737
21 d	13.0	12.4	13.4	12.7	13.3	1.022	0.302	0.265	0.623
42 d	24.1 ^a,b^	21.2 ^c^	23.8 ^a,b^	23.1 ^b^	25.0 ^a^	1.609	<0.001	<0.001	0.368
* Weight gain (g/d)*									
0–21 d	270	241	293	257	285	48	0.227	0.210	0.597
21–42 d	527 ^a,b^	418 ^c^	494 ^b^	493 ^b^	559 ^a^	40	<0.001	<0.001	0.261
0–42 d	398 ^a,b^	329 ^c^	393 ^a,b^	375 ^b^	422 ^a^	38	<0.001	<0.001	0.360
*Feed intake (g/d)*									
0–21 d	387	383	393	372	414	59	0.696	0.356	0.430
21–42 d	854 ^a,b^	733 ^c^	804 ^b^	835 ^b^	903 ^a^	61	<0.001	<0.001	0.361
0–42 d	621 ^a,b^	558 ^c^	598 ^b,c^	603 ^a,b,c^	658 ^a^	55	0.019	0.001	0.937
*Gain/feed*									
0–21 d	0.698	0.627	0.743	0.693	0.691	0.073	0.060	0.332	0.047
21–42 d	0.618 ^a^	0.573 ^b^	0.616 ^a^	0.595 ^a,b^	0.619 ^a^	0.034	0.039	0.036	0.438
0–42 d	0.643 ^a^	0.592 ^b^	0.658 ^a^	0.625 ^a,b^	0.642 ^a^	0.035	0.010	0.063	0.089
Experiment 2									
*Body weight (kg)*									
0 d	9.20	9.20	9.18	9.18	9.17	0.031	0.293	0.337	0.356
21 d	15.3	15.0	15.2	15.3	14.8	0.817	0.722	0.561	0.274
42 d	29.1	27.1	28.0	28.8	28.3	1.482	0.093	0.111	0.089
*Weight gain (g/d)*									
0–21 d	292	277	289	291	270	39	0.729	0.582	0.261
21–42 d	655 ^a^	575 ^b^	608 ^a,b^	645 ^a^	643 ^a^	57	0.048	0.021	0.165
0–42 d	473	426	449	468	456	35	0.085	0.111	0.085
*Feed intake (g/d)*									
0–21 d	416	397	397	395	389	46	0.814	0.729	0.889
21–42 d	1017	938	963	1024	977	76	0.156	0.262	0.090
0–42 d	717	668	680	710	683	50	0.256	0.484	0.169
*Gain/feed*									
0–21 d	0.718	0.717	0.740	0.749	0.718	0.070	0.824	0.932	0.265
21–42 d	0.646	0.613	0.633	0.629	0.658	0.030	0.054	0.007	0.939
0–42 d	0.665	0.640	0.663	0.660	0.671	0.028	0.273	0.063	0.434

PC = positive control; NC = negative control; NC125 = NC diet with phytase at 125 FTU/kg feed; NC250 = NC diet with phytase at 250 FTU/kg feed; NC500 = NC diet with phytase at 500 FTU/kg feed; RMSE = root mean square error; d = day. ^1^ Values in the table are LSMEANS of 8 pens per treatment for each experiment. ^2^ *p*-value for linear effect of phytase supplementation of the negative control diet. ^3^ *p*-value for quadratic effect of phytase supplementation of the negative control diet. ^a,b,c^ Values within a row with different superscripts differ significantly at *p* < 0.05 (LSD test).

**Table 3:** Effect of 6-phytase supplementation to negative control diets on Ca and P apparent total tract digestibility (ATTD, %) of the starter diets between 18–21 days ^1^.

	PC	NC	NC125	NC250	NC500	*RMSE*	*p*-Value	*L* ^2^	*Q* ^3^
Experiment 1									
P ATTD	41.2 ^a^	9.80 ^d^	21.0 ^c^	33.3 ^b^	42.0 ^a^	6.77	<0.001	<0.001	0.023
Ca ATTD	54.2 ^b,c^	51.3 ^c^	57.9 ^b^	69.3 ^a^	68.6 ^a^	4.26	<0.001	<0.001	<0.001
Experiment 2									
P ATTD	52.0 ^a^	29.1 ^d^	28.0 ^d^	40.0 ^c^	46.8 ^b^	4.36	<0.001	<0.001	0.763
Ca ATTD	48.6 ^a^	32.6 ^b^	37.5 ^b^	48.9 ^a^	45.0 ^a^	6.37	<0.001	<0.001	0.004

PC = positive control; NC = negative control; NC125 = NC diet with phytase at 125 FTU/kg feed; NC250 = NC diet with phytase at 250 FTU/kg feed; NC500 = NC diet with phytase at 500 FTU/kg feed; RMSE = root mean square error. ^1^ Values in the table are LSMEANS of 8 pens per treatment for each experiment. ^2^ *p*-value for linear effect of phytase supplementation of the negative control diet. ^3^ *p*-value for quadratic effect of phytase supplementation of the negative control diet. ^a,b,c,d^ Values within a row with different superscripts differ significantly at *p* < 0.05 (LSD test).

**Table 4:** Effect of 6-phytase supplementation to negative control diets on bone mineralization (left *Os metacarpale III*) and density (left *Os metacarpale IV*) ^1^.

	PC	NC	NC125	NC250	NC500	*RMSE*	*p-*Value	*L* ^2^	*Q* ^3^
Experiment 1									
Dry weight (g)	2.65 ^a,b^	1.85 ^c^	2.37 ^b^	2.46 ^a,b^	2.81 ^a^	0.364	<0.001	<0.001	0.185
Ash (%)	42.6 ^a^	30.1 ^d^	33.0 ^c,d^	36.0 ^b,c^	36.6 ^b^	3.34	<0.001	<0.001	0.083
Ash (g)	1.12 ^a^	0.56 ^d^	0.78 ^c^	0.88 ^b,c^	1.03 ^a,b^	0.145	<0.001	<0.001	0.095
Density (g/cm^3^)	1.56 ^a,b^	1.41 ^c^	1.49 ^b,c^	1.59 ^a^	1.62 ^a^	0.075	<0.001	<0.001	0.047
Experiment 2									
Dry weight (g)	3.24 ^a^	2.31 ^c^	2.64 ^b,c^	2.76 ^b^	2.98 ^a,b^	0.337	<0.001	<0.001	0.310
Ash (%)	43.3 ^a^	36.7 ^c^	37.6 ^c^	39.8 ^b,c^	40.9 ^a,b^	3.19	0.002	0.008	0.602
Ash (g)	1.41 ^a^	0.85 ^d^	0.99 ^c^	1.09 ^b,c^	1.22 ^b^	0.135	<0.001	<0.001	0.246
Density (g/cm^3^)	1.70	1.62	1.68	1.72	1.71	0.135	0.595	0.217	0.306

PC = positive control; NC = negative control; NC125 = NC diet with phytase at 125 FTU/kg feed; NC250 = NC diet with phytase at 250 FTU/kg feed; NC500 = NC diet with phytase at 500 FTU/kg feed; RMSE = root mean square error. ^1^ Values in the table are LSMEANS of one animal per pen (8 pens per treatment) for each experiment. ^2^ *p*-value for linear effect of phytase supplementation of the negative control diet. ^3^ *p*-value for quadratic effect of phytase supplementation of the negative control diet. ^a,b,c,d^ Values within a row with different superscripts differ significantly at *p* < 0.05 (LSD test).

**Table 5:** Calcium (Ca) and phosphate (P) concentration and alkaline phosphatase activity in blood plasma of piglets fed a positive control diet (PC), a negative control diet (NC) with limiting levels of Ca and P, or an NC diet supplemented with 500 FTU/kg of phytase for 42 days ^1^.

	PC	NC	NC500	*RMSE*	*p-*Value
Experiment 1					
Calcium (mmol/L)	2.45	2.31	2.46	0.158	0.145
Phosphate (mmol/L)	2.79 ^a^	1.79 ^b^	2.55 ^a^	0.347	<0.001
Ca:P ratio	0.89 ^b^	1.33 ^a^	0.98 ^b^	0.190	0.001
Alkaline Phosphatase (I.U./L)	414 ^b^	1001 ^a^	506 ^b^	227.5	<0.001
Experiment 2					
Calcium (mmol/L)	2.82	2.83	2.88	0.194	0.807
Phosphate (mmol/L)	2.95 ^a^	2.19 ^b^	3.18 ^a^	0.321	<0.001
Ca:P ratio	0.96 ^b^	1.34 ^a^	0.92 ^b^	0.176	<0.001
Alkaline Phosphatase (I.U./L)	173 ^b^	225 ^a^	168 ^b^	36.5	0.013

PC = positive control; NC = negative control; NC500 = NC diet with phytase at 500 FTU/kg feed; RMSE = root mean square error. ^1^ Values in the table are LSMEANS of one animal per pen (8 pens per treatment) for each experiment. ^a,b^ Values within a row with different superscripts differ significantly at *p* < 0.05 (LSD test).

## Data Availability

Not applicable.
